# Algorithms for locating extremely conserved elements in multiple sequence alignments

**DOI:** 10.1186/1471-2105-10-432

**Published:** 2009-12-18

**Authors:** Huei-Hun E Tseng, Martin Tompa

**Affiliations:** 1 Department of Computer Science and Engineering, University of Washington, Box 352350, Seattle, WA, 98195-2350, USA; 2 Department of Genome Sciences, University of Washington, Seattle, WA, 98195-5065, USA

## Abstract

**Background:**

In 2004, Bejerano *et al*. announced the startling discovery of hundreds of "ultraconserved elements", long genomic sequences perfectly conserved across human, mouse, and rat. Their announcement stimulated a flurry of subsequent research.

**Results:**

We generalize the notion of ultraconserved element in a natural way from extraordinary human-rodent conservation to extraordinary conservation over an arbitrary set of species. We call these "Extremely Conserved Elements". There is a linear time algorithm to find all such Extremely Conserved Elements in any multiple sequence alignment, provided that the conservation is required to be across all the aligned species. For the general case of conservation across an arbitrary subset of the aligned species, we show that the question of whether there exists an Extremely Conserved Element is *NP*-complete. We illustrate the linear time algorithm by cataloguing all 177 Extremely Conserved Elements in the currently available 44-vertebrate whole-genome alignment, and point out some of the characteristics of these elements.

**Conclusions:**

The *NP*-completeness in the case of conservation across an arbitrary subset of the aligned species implies that it is unlikely an efficient algorithm exists for this general case. Despite this fact, for the interesting case of conservation across all or most of the aligned species, our algorithm is efficient enough to be practical. The 177 Extremely Conserved Elements that we catalog demonstrate many of the characteristics of the original ultraconserved elements of Bejerano *et al*.

## Background

In 2004, Bejerano *et al*. [1] made the startling discovery that there are hundreds of long genomic sequences extraordinarily conserved across human, mouse, and rat, most of them in noncoding regions and some of them very distant from the nearest human gene. They defined an "ultraconserved element" to be at least 200 consecutive alignment columns, 100% of these columns perfectly conserved in human, mouse, and rat. They reported 481 such elements across the human genome, exclusive of rRNA genes. They also reported incidentally that some fraction (99%, 97%, and 67%, respectively) of these ultraconserved elements are also well conserved in dog, in chicken, and in fugu, though with fewer than 100% of the columns perfectly conserved in these other species even in this fraction of elements.

Their introduction of ultraconserved elements stimulated a flurry of subsequent research. Derti *et al*. [2] compiled lists of similarly perfectly conserved elements in human-mouse-dog and in human-chicken, and observed a surprisingly small overlap in these three sets of ultraconserved elements. Sakuraba *et al*. [3] compiled a list of elements each more than 500 bp long and each more than 95% identical between human and mouse, and observed that only 9-14% of these have conserved matches in fishes. Visel *et al*. [4] showed that the noncoding ultraconserved elements of Bejerano *et al*. [1] were only a subset of elements under similar constraint and with similar regulatory function. They generated this larger set of elements using Gumby scores [5], and showed that this larger set of noncoding ultraconserved elements is highly enriched in mouse enhancer activity. Rather than using percent of perfectly conserved columns, Siepel *et al*. [6] incorporated the phylogeny into the measure of conservation by using a phylogenetic hidden Markov model, and defined Highly Conserved Elements to be those with the highest log-odds scores, measuring how much more likely they are to be generated in the conserved than the nonconserved state of the model.

Most of these works defined their conserved elements in terms of conservation across just 2 or 3 species, Siepel *et al*. [6] being an exception. In this paper we extend the notion of ultraconserved elements in a very natural way to an arbitrarily large collection of species.

Suppose that you are given a whole-genome multiple sequence alignment, such as the current 44-vertebrate whole-genome alignment available through the UCSC Genome Browser [7]. You are interested in finding long regions of this alignment that are extraordinarily well conserved across all or most of the 44 species, in the spirit of generalizing the notion of ultraconserved elements of Bejerano *et al*. [1]. For concreteness in this introduction, let us say that you want to identify all regions of at least 100 consecutive alignment columns such that, for some subset *S *of at least 40 of the 44 species, at least 80% of the columns in this region are perfectly conserved (i.e., contain the same nucleotide) across all the species in *S*. Because the 44-vertebrate whole-genome alignment occupies approximately 250 gigabytes of memory, algorithmic efficiency is a concern.

In the next section, we consider the generalization of this problem instance to arbitrary alignments, any number of columns, any minimum cardinality |*S*|, and any percentage of conserved columns. We call such well conserved regions "Extremely Conserved Elements". We present a linear time algorithm for finding all such elements, provided the subset *S *is the entire set of aligned species. For an arbitrary subset *S*, we demonstrate that the problem is *NP*-complete [8] and hence it is unlikely that there is an efficient algorithm for finding Extremely Conserved Elements in this general case.

As an illustration of the linear time algorithm, we present results for the concrete instantiation of the problem given above. In the current 44-vertebrate whole-genome alignment, there are 177 elements, each with at least 100 alignment columns and each perfectly conserved across the same 40 or more vertebrates in at least 80% of its columns. The longest such element is 355 columns long and occurs 60 Kbp from the nearest gene on human chromosome 19, perfectly conserved in 80% of its columns across 41 of the species, missing only gorilla, shrew, and lamprey. There is also a region 70 bp long, 7 Kbp upstream of the *FOXB2 *gene on human chromosome 9, with 90% of its columns perfectly conserved across all 44 vertebrates.

## Results and discussion

### Problem statement and algorithms

We begin with a precise formulation of the *Extremely Conserved Element *problem:

**Inputs**: *m *× *n *alignment matrix *M *with entries from {A, C, G, T, -}, integer *s *≤ *m*, integer *t *≤ *n*, and real number 0 <*c *≤ 1.

**Problem**: Determine if *M *has a subset *S *of rows, a subset *T *of consecutive columns (ignoring columns that contain the gap character "-" in every row of *S*), and a subset *U *of *T*, with |*S*| ≥ *s*, |*T*| ≥ *t*, and |*U*| ≥ *c*|*T*| such that, in the matrix *M *restricted to *S *× *U*, every column is perfectly conserved (that is, all the elements in that column are equal). Note that neither *S *nor *U *need be consecutive.

For example, the illustrative problem in the introduction is the version of the Extremely Conserved Element problem with *m *= 44, *n *≈ 3.8 × 10^9^, *s *= 40, *t *= 100, and *c *= 0.8.

The condition that every column in *S *× *U *be perfectly conserved is overly simplistic. More realistically, the conservation measure should depend on the phylogeny relating the species, as does, for example, the measure used by phastCons [6]. Suppose that *F *is any conservation scoring function (that may depend on the phylogeny). The algorithm of Theorem 1 is easily generalized to the problem in which every column in *S *× *U *must have *F *exceeding some threshold τ.

**Theorem 1**: If *s *= *m*, the Extremely Conserved Element problem can be solved in time O(*mn*). In fact, the maximum value of *t *can be determined in this time.

**Proof**: Assume without loss of generality that no column contains the gap character "-" in every row. For 1 ≤ *i *≤ *n*, let *q*_*i *_= 1 if column *i *is perfectly conserved, and *q*_*i *_= 0 otherwise. The result then follows from Theorem 2.   □

**Theorem 2**: Given *q *∈ {0,1}^*n *^and 0 <*c *≤ 1, there is an O(*n*) time algorithm that maximizes *j *- *i *subject to the condition that *q*_*i*__+1 _... *q*_*j *_contains at least *c*(*j *- *i*) 1's.

**Proof**: A variety of linear time algorithms for this problem have appeared in the literature [9-11], although this is the first time it has been applied to multiple sequence alignments on a gigabyte scale. We give here a new and simpler algorithm due to Eddie Grove and Benno Schwikowski (personal communication). For 0 ≤ *i *≤ *n*, let

Let *x *be the number of 1's in *q*_*i*__+1 _... *q*_*j*_. Then

Thus, *r*_*j *_≥ *r*_*i *_if and only if *q*_*i*__+1 _... *q*_*j *_has at least *c*(*j *- *i*) 1's, so the objective is to maximize *j *- *i *subject to *r*_*j *_≥ *r*_*i*_.

For 0 ≤ *i *≤ *n*, let *X*_*i *_= min(*r*_0_, *r*_1_, ..., *r*_*i*_) and *Y*_*i *_= max(*r*_*i*_, *r*_*i*__+1_, ..., *r*_*n*_). *X *and Y are each nonincreasing sequences. We claim that the objective above is equivalent to maximizing *j *- *i *subject to *Y*_*j *_≥ *X*_*i*_: If *r*_*j *_≥ *r*_*i*_, then *Y*_*j *_= max(*r*_*j*_, *r*_*j*__+1_, ..., *r*_*n*_) ≥ *r*_*j *_≥ *r*_*i *_≥ min(*r*_0_, *r*_1_, ..., *r*_*i*_) = *X*_*i*_. If *Y*_*j *_≥ *X*_*i *_and *X*_*i *_<*X*_*i*__-1 _and *Y*_*j *_>*Y*_*j*__+1_, then *r*_*i *_= *X*_*i *_and *r*_*j *_= *Y*_*j*_, so *r*_*j *_≥ *r*_*i*_. In particular, (*i*, *j*) maximizes *j *- *i *subject to *r*_*j *_≥ *r*_*i *_if and only if (*i*, *j*) maximizes *j *- *i *subject to *Y*_*j *_≥ *X*_*i*_. Since *X *and *Y *are sorted, the latter can be found in linear time: merge *X *and *Y*, breaking ties by taking elements from *Y *first. Then identify the maximum *j *- *i *such that *Y*_*j *_and *X*_*i *_are adjacent (in this order) in the sorted list.   □

In fact, in the proof of Theorem 2, *any *pair (*i*, *j*) such that *Y*_*j *_and *X*_*i *_are adjacent (in this order) in the sorted list corresponds to a maximal interval *q*_*i*__+1 _... *q*_*j *_that contains at least *c*(*j *- *i*) 1's. Thus, all such maximal intervals can be found in linear time.

The dual of Theorem 2, maximizing *c *subject to a lower bound on *j *- *i*, also can be accomplished in linear time [12,13]. This implies that, for *s *= *m*, the maximum value of *c *in the Extremely Conserved Element problem can also be determined in time O(*mn*).

For arbitrary *S*, the following theorem provides another special case in which the problem can be solved efficiently.

**Theorem 3**: If *c *= 1, the Extremely Conserved Element problem can be solved in polynomial time. In fact, the maximum value of *s *can be determined in this time.

**Proof**: For every choice *T *of at least *t *consecutive columns, sort the rows of *T *lexicographically and look for *s *identical rows, with at least *t *nongap characters each, in this sorted list.   □

However, for the general case, the following theorem shows that it is unlikely that there is an efficient algorithm [8].

**Theorem 4**: The general Extremely Conserved Element problem is *NP*-complete, even if *t *= *n *and *M*'s entries are all either A or T.

**Proof**: The reduction used is very similar to the reduction from Clique to Balanced Complete Bipartite Subgraph [14].

Let (*G*, *K*) be an instance of the Clique problem, where *G *= (*V*, *E*) is an undirected graph and *K *is an integer. Assume without loss of generality that *K *< |*V*| - 2. Consider the bipartite graph *B *= ((*X*, *Y*), *F*), where *X *= *V*, *Y *= *E*, and *F *= {(*v*, *e*) | *v *∉ *e*}. Let *M *be the |*X*| × |*Y*| adjacency matrix of *B*, where *M_ij_*__= A if (*X*_*i*_, *Y*_*j*_) ∈ *F *and *M*_*ij *_= T otherwise. Let *s *= |*V*| - *K*, *t *= |*E*|, and *c *= /|*E*|. We will show that *G *has a *K*-clique if and only if *M *has the appropriate Extremely Conserved Element.

Suppose that *C *⊆ *V *is a *K*-clique of *G*. Choose *S *= *V *- *C *and *U *= {{*u*, *v*} | *u *∈ *C *and *v *∈ *C*}. |*S*| = |*V*| - *K *and |*U*| =  and, in *B*, every vertex in *S *is adjacent to every vertex in *U*. Hence, the submatrix of *M *restricted to *S *× *U *contains only the character A.

Conversely, suppose that *M *has a (|*V*| - *K*) ×  submatrix *M' *each of whose columns is perfectly conserved. Since each vertex *v *in *Y *is not adjacent to exactly 2 vertices in *X*, it is impossible for *v*'s column of *M' *to consist only of the character T when |*V*| - *K *> 2, as is the case. Hence, *M' *contains only the character A. That is, there are subsets *S *of *X *and *U *of *Y *that form a complete bipartite subgraph of *B*, with |*S*| = |*V*| - *K *and |*U*| = . Every vertex {*u*, *v*} ∈ *U *must satisfy *u *∈ *V *- *S *and *v *∈ *V *- *S *since, in *B*, {*u*, *v*} is adjacent to every vertex in *S*. But |*V *- *S*| = *K*, so the  vertices in *U *correspond to the edges of a *K*-clique *V *- *S *in *G*.   □

### Catalog of Extremely Conserved Elements

As an illustration of Theorem 1, we present results for the concrete instantiation of the problem given in the introduction. In the current 44-vertebrate whole-genome MULTIZ alignment (human genome assembly UCSC hg18, March 2006) available through the UCSC Genome Browser [7], we identify all Extremely Conserved Elements with at least 100 consecutive alignment columns such that, for some subset *S *of at least 40 species including human, at least 80% of the columns in this region are perfectly conserved across all the species in *S*. (If some column contains the gap character "-" in all species of *S*, that column is ignored, contributing neither to the count of 100 columns nor to the percent perfectly conserved.) We refer to such elements as *EC(40, 100, 0.8) elements*.

Despite the *NP*-completeness demonstrated in Theorem 4, the algorithm of Theorem 1 still allows a feasible solution to this particular instantiation of the general Extremely Conserved Element problem. The simple reason is that  = 149,986 is not an impossibly large number of combinations on which to run the linear time algorithm. Had we asked for conservation across only 22 rather than 40 species, the computation would have been prohibitive, because  > 2 × 10^12^. In addition, there are two novel filters described in Methods that make the application to whole-genome alignments feasible.

In the current 44-vertebrate whole-genome alignment, our algorithm produces 177 EC(40, 100, 0.8) elements. A complete listing of these elements is given in additional files. Additional file 1 is a spreadsheet that shows each EC(40, 100, 0.8) element's human genome coordinates, its length, percent identity, names of missing species, name of the human gene in which it resides (if appropriate), names of and distances to the two nearest neighboring genes, and overlap with previously identified conserved elements [1,4,6]. Additional file 2 shows the exact alignment for each EC(40, 100, 0.8) element. Additional file 3 shows the single longest extremely conserved element that has at least 90% of its columns perfectly conserved across all 44 vertebrate species. It is 70 bp long. (In Additional files 1 and 2, the lengths of some of the elements are shown as slightly less than 100 columns. The reason is that we have removed from every element those columns at each end that are not perfectly conserved. Any of these elements can be padded on either end to 100 columns while still maintaining at least 80% identical columns.)

For the 240 human genes either containing an EC(40, 100, 0.8) element, or immediately upstream or downstream from that element if it is between genes, we investigated enrichment for biological process GO terms, using WebGestalt [15]. The results are shown in Table 1. These enrichments are in accord with previous human-rodent studies [1,4], which reported significant functional enrichment for genes involved in regulation of transcription, DNA binding, development, and nervous system development.

**Table 1:** Enrichment for biological process GO terms of the 240 human genes containing or neighboring EC(40, 100, 0.8) elements.

*GO category*	*observed genes*	*expected number*	*p-value*
regulation of transcription	70	25.68	3.30 × 10^-17^
development	43	22.36	1.04 × 10^-5^
organ development	18	6.74	1.34 × 10^-4^
nervous system development	16	5.62	1.56 × 10^-4^

For the genes containing the 25 EC(40, 100, 0.8) elements that overlap human coding exons, there is functional enrichment for RNA processing (*p*-value = 2.20 × 10^-4^). This is consistent with the reported findings for genes containing ultraconserved elements that overlap coding exons, which show significant functional enrichment for RNA binding and regulation of splicing [1].

**Figure 1:**
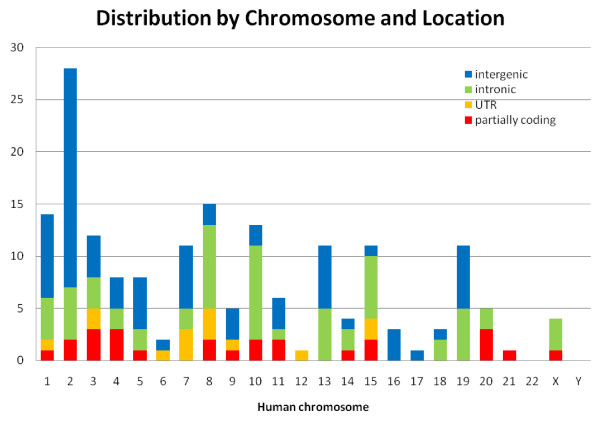
**Distribution of 177 EC(40, 100, 0.8) elements by human chromosome and location with respect to human genes. See text for the explanation of location labels**.

Figure 1 shows the distribution of the 177 EC(40, 100, 0.8) elements by human chromosome and location with respect to human genes. If an element overlaps a human coding exon (in some splice form), it is called "partially coding". If not and it is entirely contained within an intron between two coding exons (in some splice form), it is called "intronic". If it is between the annotated transcription start and stop sites of a gene but is neither partially coding nor intronic, it is called "UTR". (Most of these elements are actually contained in a UTR intron rather than overlapping the mature UTR.) If it is entirely contained between two genes (in all splice forms), it is called "intergenic". In total there are 25 partially coding, 14 UTR, 61 intronic, and 77 intergenic EC(40, 100, 0.8) elements; these account for 14%, 8%, 34%, and 44%, respectively, of all 177 elements. None of the elements overlap annotated rRNA, tRNA, or other annotated noncoding RNA genes. Of the 77 intergenic elements, 65 (37% of all 177 elements) are more than 10 Kbp from the nearest human gene and 42 (24% of all 177 elements) are more than 100 Kbp from the nearest gene. These percentages are somewhat greater than the corresponding percentages (29% over 10 Kbp and 18% over 100 Kbp) for intergenic ultraconserved elements [1].

As was observed for ultraconserved elements [1], it is quite common to see three to eight EC(40, 100, 0.8) elements clustered within the same gene or within the same intergenic region (see Additional file 1). As one extreme example, eight occur within the introns and another two upstream of the human gene *ZFPM2 *on chromosome 8. ZFPM2 is a zinc finger transcription factor that modulates the activity of GATA family proteins, which are important regulators of cardiogenesis. None of these ten *ZFPM2 *EC(40, 100, 0.8) elements overlap ultraconserved elements of Bejerano *et al*. [1], and three of them do not even overlap the larger set of constrained human-rodent elements of Visel *et al*. [4], so are novel.

The longest EC(40, 100, 0.8) element found is 355 columns long and is perfectly conserved in 80% of its columns across 41 of the species, missing only gorilla, shrew, and lamprey. It occurs 60 Kbp downstream of the nearest gene on human chromosome 19. But interestingly it is 297 Kbp upstream of its other neighboring gene *ZNF536*, which has two other upstream and three intronic EC(40, 100, 0.8) elements. ZNF536 is a zinc finger protein, expressed in the developing central nervous system, that negatively regulates neuron differentiation [16]. Only two of these six EC(40, 100, 0.8) elements overlap ultraconserved elements of Bejerano *et al*. [1].

Note, though, that the distribution of the lengths of EC(40, 100, 0.8) elements is not significant since, as pointed out in Methods, our algorithm sacrifices length in preference for number of species in the element. As one extreme example, there are abutting elements at human hg18 coordinates chrX:24826152-24826332 and chrX:24826333-24826597 of lengths 188 and 276, respectively. The first is missing alpaca, sloth, medaka, and lamprey, while the second is missing only alpaca, medaka, and lamprey. Had the algorithm not prioritized number of species over length, it would have produced a single EC(40, 100, 0.8) element of length 464, which would have been longer than the current longest element. These two current elements are two out of three EC(40, 100, 0.8) elements that occur in an intron of *POLA1*, which encodes the DNA polymerase alpha catalytic subunit.

Lamprey, the species most distant from the mammals and also a low-coverage genome assembly, is missing from 170 of the 177 EC(40, 100, 0.8) elements. The species with the next highest numbers are gorilla (missing from 41), cat (missing from 35), and zebrafish (missing from 30). Like lamprey, gorilla and cat are low-coverage genome assemblies. On the other hand, fugu is missing from only 4 of the 177 EC(40, 100, 0.8) elements, zebra finch is missing from only 3, chicken is missing from only 2, and lizard is missing from only 1. These figures suggest that depth of sequencing is a greater determinant of inclusion in extremely conserved elements than is evolutionary distance, and also suggest that additional extremely conserved elements will emerge as the quality of genome sequences increases.

47 (27%) of the 177 EC(40, 100, 0.8) elements overlap an ultraconserved element of Bejerano *et al*. [1]. 117 (66%) overlap the larger set of constrained human-rodent elements of Visel *et al*. [4]. These overlaps confirm the fact that we have identified significant conserved elements that cannot be found by applying a more stringent conservation criterion to a smaller collection of species such as human and rodents. All 177 EC(40, 100, 0.8) elements overlap phastCons Highly Conserved Elements [6] (as computed in the UCSC 44-vertebrate alignment) with phastCons log-odds scores each exceeding 1300. Details of all these overlaps are shown in Additional file 1. The phastCons Highly Conserved Elements have the realistic advantage of taking the phylogeny and branch lengths into account, which our Extremely Conserved Elements do not. However, because their log-odds scores favor longer alignments, phastCons Highly Conserved Elements tend to be much longer than EC(40, 100, 0.8) elements, with less intense concentration of extremely conserved columns. There are 12,749 phastCons Highly Conserved Elements with log-odds score at least 1300, of which only a certain 177 contain within them EC(40, 100, 0.8) elements. The average length of these 12,749 elements is 500 bp, with many of them several kilobasepairs long, whereas the average EC(40, 100, 0.8) element is less than 133 bp long. There is no obvious way of querying phastCons Highly Conserved Elements so as to identify those with such intense concentration of extremely conserved columns. For instance, if you restrict attention to those Highly Conserved Elements with log-odds score at least 1300 and length at most 400 bp, this long list would contain only 43 of the 177 EC(40, 100, 0.8) elements; the remaining 134, though as extremely conserved, occur within longer Highly Conserved Elements.

Of the genes containing EC(40, 100, 0.8) elements that do not contain nor are adjacent to either ultraconserved elements of Bejerano *et al*. [1] nor the larger set of constrained human-rodent elements of Visel *et al*. [4], three are particularly notable because their functions are closely related to the GO term enrichments discussed near the beginning of this section. *NLGN1 *on human chromosome 3 contains an intronic EC(40, 100, 0.8) element and is involved in nervous system development. In particular, this gene encodes a member of a family of neuronal cell surface proteins that may be involved in the formation and remodeling of central nervous system synapses. *WHSC1L1 *on human chromosome 8 contains a partially coding EC(40, 100, 0.8) element and is involved both in regulation of transcription and in cell differentiation. More specifically, this gene encodes a histone methyltransferase that preferentially methylates K4 and K27 of histone H3, which are epigenetic tags for transcriptional regulation. *RBM5 *on human chromosome 3 contains a partially coding EC(40, 100, 0.8) element and, like many other genes with such coding elements, is involved in RNA processing. In particular, this gene encodes a component of the spliceosome A complex and regulates alternative splicing of a number of mRNAs, including FAS and CASP2/caspase-2 in the apoptosis process. In the case of FAS, it promotes exclusion of exon 6, producing a soluble form of FAS that inhibits apoptosis. In the case of CASP2/caspase-2, it promotes exclusion of exon 9, producing a catalytically active form of CASP2/Caspase-2 that induces apoptosis. These three genes, *NLGN1*, *WHSC1L1*, and *RBM5*, are just some of the genes containing extremely conserved elements that were not uncovered in previous studies of ultraconservation.

## Conclusions

The notion of ultraconserved element was introduced by Bejerano *et al*. [1] to point out that there are certain long genomic sequences with extreme conservation between humans and rodents. We generalized their definition in a natural way that allows one to look for such extreme conservation over a much larger collection of species. We presented a linear time algorithm (Theorem 1) to find all such maximal length Extremely Conserved Elements, provided that one insists on conservation across all the aligned species. In contrast, we showed that, for the general case of conservation across an arbitrary subset of the species, the question of whether there is an Extremely Conserved Element is *NP*-complete (Theorem 4), so it is unlikely an efficient algorithm exists for this general case [8]. This is true even in the special case of a gapless multiple sequence alignment.

Finally, we illustrated the linear time algorithm by identifying all Extremely Conserved Elements with *s *= 40, *t *= 100, and *c *= 0.8 in the currently available 44-vertebrate whole-genome alignment. The resulting 177 EC(40, 100, 0.8) elements demonstrate many of the characteristics of the original ultraconserved elements [1].

## Methods

In this section we describe two novel filters that make the application of Theorem 1 to whole-genome alignments feasible. Whole-genome MULTIZ alignments are divided into "alignment blocks" (Blanchette *et al*. [17]), where each block contains some subset of the species aligned to some region of the reference genome (human, in our case). A common reason for a boundary between alignment blocks is that some species enters or leaves the alignment at that boundary point. Our first filter simply reads through all the alignment blocks, retaining only those containing at least 40 species.

In theory it is possible that an EC(40, 100, 0.8) element could span an arbitrary alignment block *B *consisting of *b *columns that was discarded by Filter 1, if there were enough perfectly conserved columns among 40 species before and after *B *to make up for *b *unconserved columns. However, if *b *≥ 50, this would mean that there is also an EC(40, 100, 0.8) element on one side of *B *or the other. Therefore, we next merge alignment blocks as long as the separation between those blocks on the human genome is less than 50 bp; a separation greater than this signals the end of the current merged block and the beginning of a new one. More specifically, if *A *is the current merged block, *C *is the next alignment block following *A *that was retained after Filter 1, and *A *and *C *are separated by human sequence *B *with |*B*| < 50, we append *BC *to the end of *A *and insert the gap character "-" in row *s *of any alignment column of *B *or *C *that was missing species *s*.

We are now in a position to describe Filter 2, which is run on each merged block independently. For the *i-*th alignment column of the current merged block, let *q*_*i *_= 1 if column *i *has 40 or more equal characters (including "-"), and *q*_*i *_= 0 otherwise. Run the algorithm of Theorem 2 on the resulting string *q *and *c *= 0.8 to identify the maximum length substring *d *of *q *that contains at least 80% 1's. Such a substring may not correspond to an EC(40, 100, 0.8) element, because there is no assurance that the set of 40 species is the same for each column, but any EC(40, 100, 0.8) element must be contained in such a substring of length at least 100. Filter 2, therefore, discards any merged block for which |*d*| < 100.

On each merged block remaining after Filter 2, we now exhaustively try all combinations of at least 40 species and run the algorithm of Theorem 1 on each combination. In order to maximize the number of species in each EC(40, 100, 0.8) element, we enumerate the combinations in decreasing order from 44 species down to 40 species. Whenever we find an EC(40, 100, 0.8) element, we remove the longest such element for the current combination of species, and then run Filter 2 again on each of the two remaining pieces of the merged block, discarding either such piece if it fails to pass Filter 2. Note that this may sacrifice a longer EC(40, 100, 0.8) element in preference for one with a greater number of species if these elements overlap.

Table 2 shows the effectiveness of the two filters and the total running time of each of the three phases of the algorithm. Filter 1 removed all but 0.25% of its alignment columns and, of the remaining columns, Filter 2 removed all but 2.3%. Since the full exhaustive algorithm on these remaining 220,646 alignment columns still required 47 hours of computing time, it is clear that the two filters are imperative.

**Table 2:** Effectiveness of the algorithmic filters in reducing the number of alignment columns to process.

	*Filter 1*	*Filter 2*	*Exhaustive algorithm*
*Columns to process*	3,843,856,747	9,606,738	220,646
*Time (in hours)*	2.6	0.1	47.3

The running time of each phase of the algorithm (run on an Intel Xeon Quad Core X7350 2.93 GHz with 132 GB RAM) is also shown.

The source code was written in Python and is available from the authors.

## Authors' contributions

Both authors contributed equally and read and approved the final manuscript.

## Supplementary Material

Additional file 1 **EC40_100_80**. A spreadsheet showing each EC(40, 100, 0.8) element's human genome coordinates, its length, percent identity, number of conserved species, names of missing species, name of the human gene in which it resides (if appropriate), names of and distances to the two nearest neighboring genes, and overlap with previously identified conserved elements [1,4,6]. Click here for file

Additional file 2 **EC40_100_80**. An alignment file showing the exact alignment for each EC(40, 100, 0.8) element. Click here for file

Additional file 3 **EC44_70_90**. An alignment file showing the single longest extremely conserved element that has at least 90% of its columns perfectly conserved across all 44 vertebrate species. Click here for file
